# Prophylactic Application of Closed-Incision Negative Pressure Therapy in a High-Risk Emergency Laparotomy: A Case Report

**DOI:** 10.7759/cureus.86887

**Published:** 2025-06-27

**Authors:** Swapnil Tripathi, Avinash Ray, Tanya Dhawan, Mohammed Athif Khan

**Affiliations:** 1 General Surgery, Worcestershire Acute Hospital NHS Trust, Worcester, GBR; 2 Community Medicine, Army Medical Corps, Indian Air Force, Gwalior, IND; 3 Orthopaedics and Trauma, United Lincolnshire Hospital NHS Trust, Boston, GBR; 4 Medicine, Worcestershire Acute Hospital NHS Trust, Worcester, GBR

**Keywords:** cinpt, closed-incision negative pressure therapy, emergency laparotomy, surgical site infection, wound management

## Abstract

Surgical site infections (SSIs) and wound dehiscence are common complications following laparotomy, particularly in patients with comorbidities such as diabetes and obesity. Closed-incision negative pressure therapy (ciNPT) is increasingly recognised for its potential to improve surgical site outcomes by reducing infection rates and promoting wound healing.

This case report describes the use of ciNPT in a patient with a high-risk surgical incision, highlighting its clinical benefits and practical application. We report the case of a 68-year-old obese female with type 2 diabetes mellitus who underwent emergency laparotomy for perforated diverticulitis. ciNPT with novel foam dressings was used postoperatively. The wound healed without infection or dehiscence. The patient experienced minimal discomfort and was discharged on postoperative day six.

The therapy demonstrated effective management of the incision site, reduced postoperative complications, and enhanced patient recovery. Imaging studies further illustrate the wound healing process under ciNPT. This report underscores the value of ciNPT as an adjunctive tool in surgical wound management in high-risk abdominal surgeries to reduce complications and facilitate recovery.

## Introduction

Surgical site infections (SSIs) remain among the most common postoperative complications, particularly in abdominal surgeries, leading to increased morbidity, prolonged hospital stays, and healthcare costs [1,2]. Patients with obesity, diabetes mellitus, or undergoing emergency procedures are especially vulnerable [3]. Emergency colorectal procedures and surgeries involving perforation or peritonitis are particularly prone to infection.

Closed-incision negative pressure therapy (ciNPT) has emerged as a novel strategy to enhance healing and reduce wound complications in high-risk patients. The therapy involves the application of a sterile, sealed dressing connected to a negative pressure unit, aiming to reduce tension at the incision site, remove exudate, and improve perfusion [4,5]. It also creates a sealed environment that protects the wound from external contamination.

ciNPT is most commonly indicated in patients at high risk of wound complications, including those undergoing abdominal, vascular, orthopaedic, and cardiothoracic surgeries. Its use is particularly prevalent in obese and diabetic patients, and in procedures with high contamination risk or complex wound closure. Several clinical guidelines and studies have supported its role in reducing postoperative complications in these contexts.

This report presents the successful use of ciNPT with novel foam dressings in a high-risk patient undergoing emergency laparotomy, contributing further evidence for its routine use in select surgical populations.

## Case presentation

**Figure 1:**
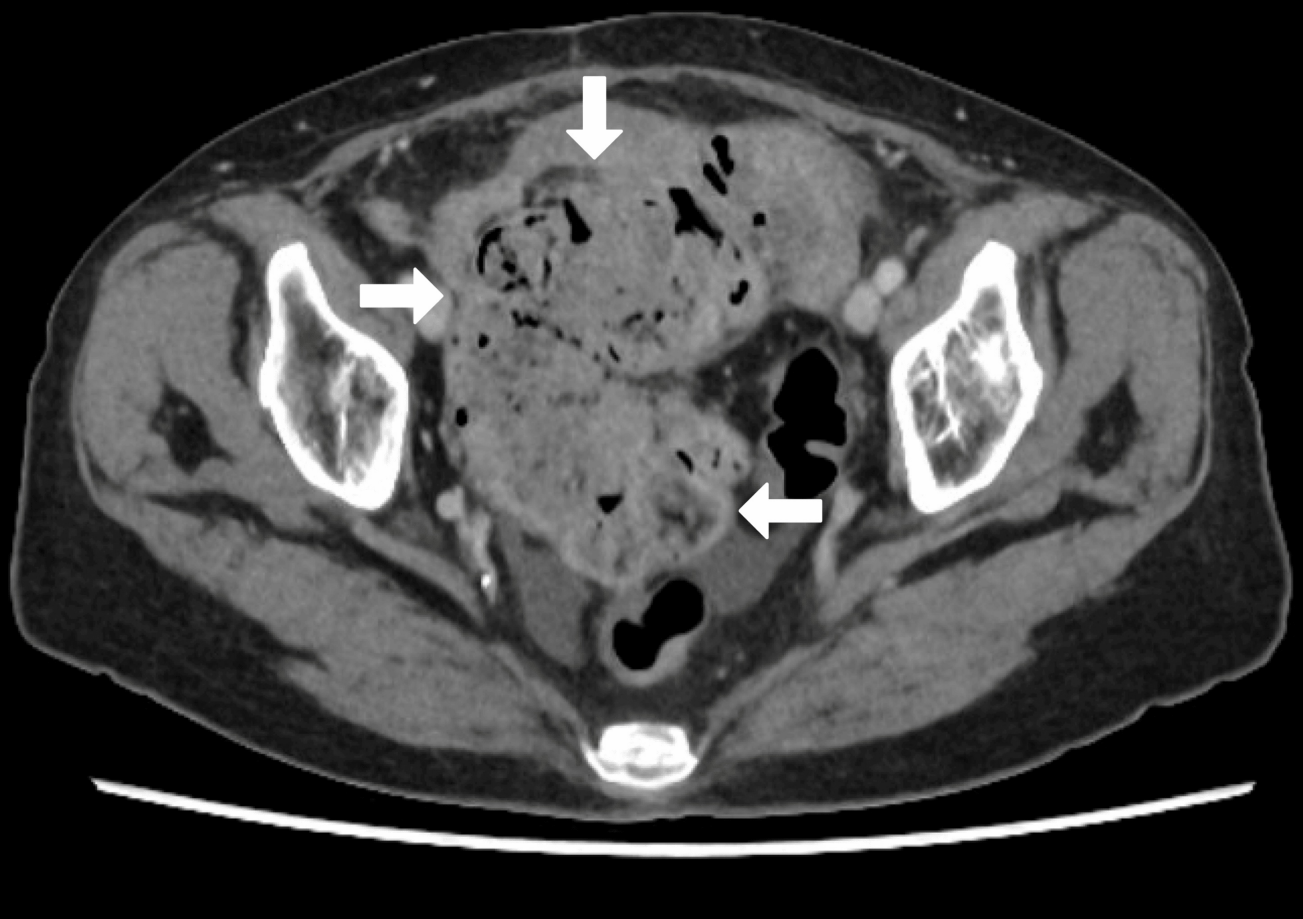
CT Abdomen & Pelvis (Axial View) Perforated sigmoid colon just above the promontory with limited contamination in pelvis, the sigmoid colon severely inflamed and full of hard stool with a 2 cm necrotic patch at the mid sigmoid, site of perforation.

**Figure 2:**
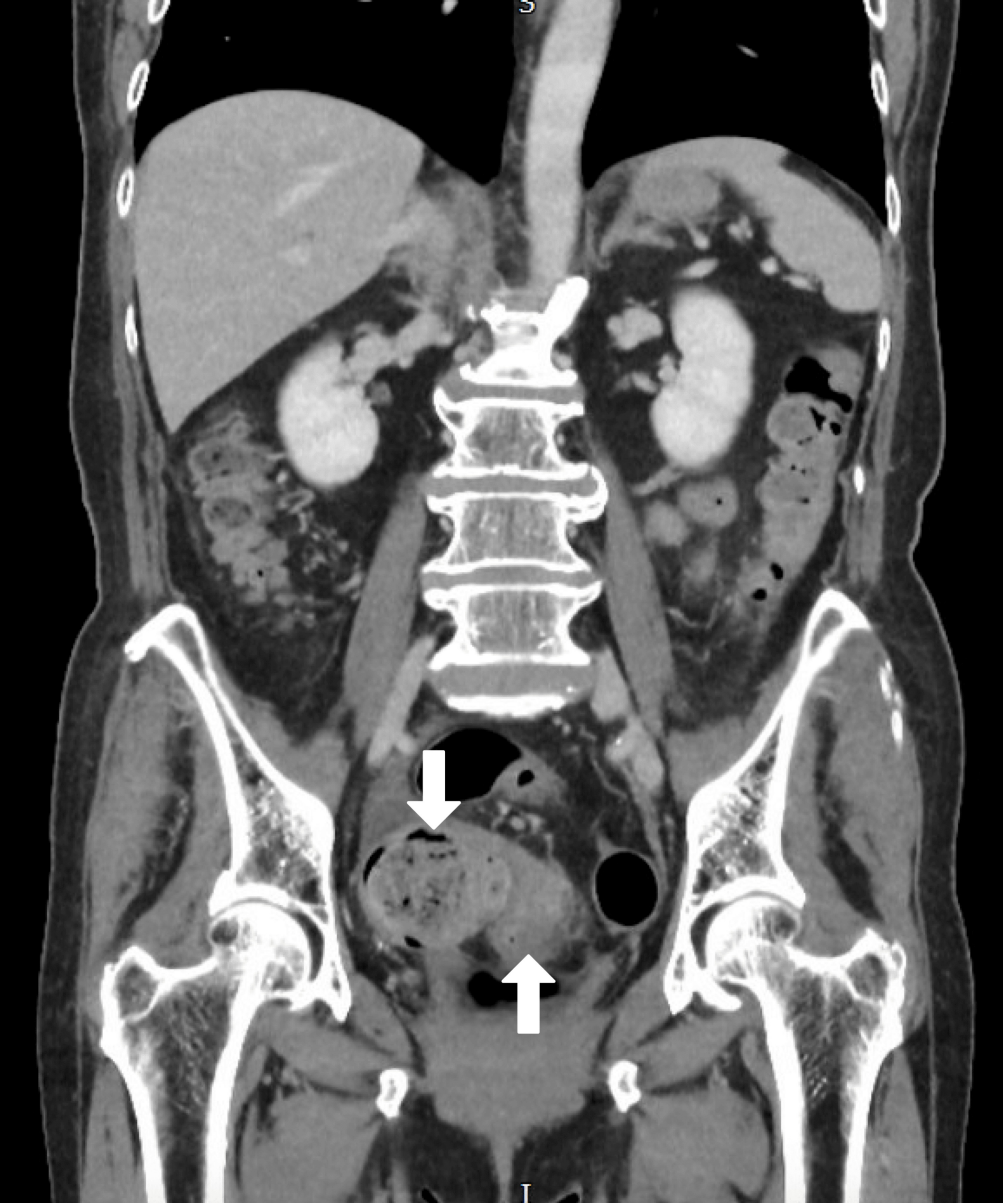
CT Abdomen & Pelvis (Coronal View)

**Figure 3:**
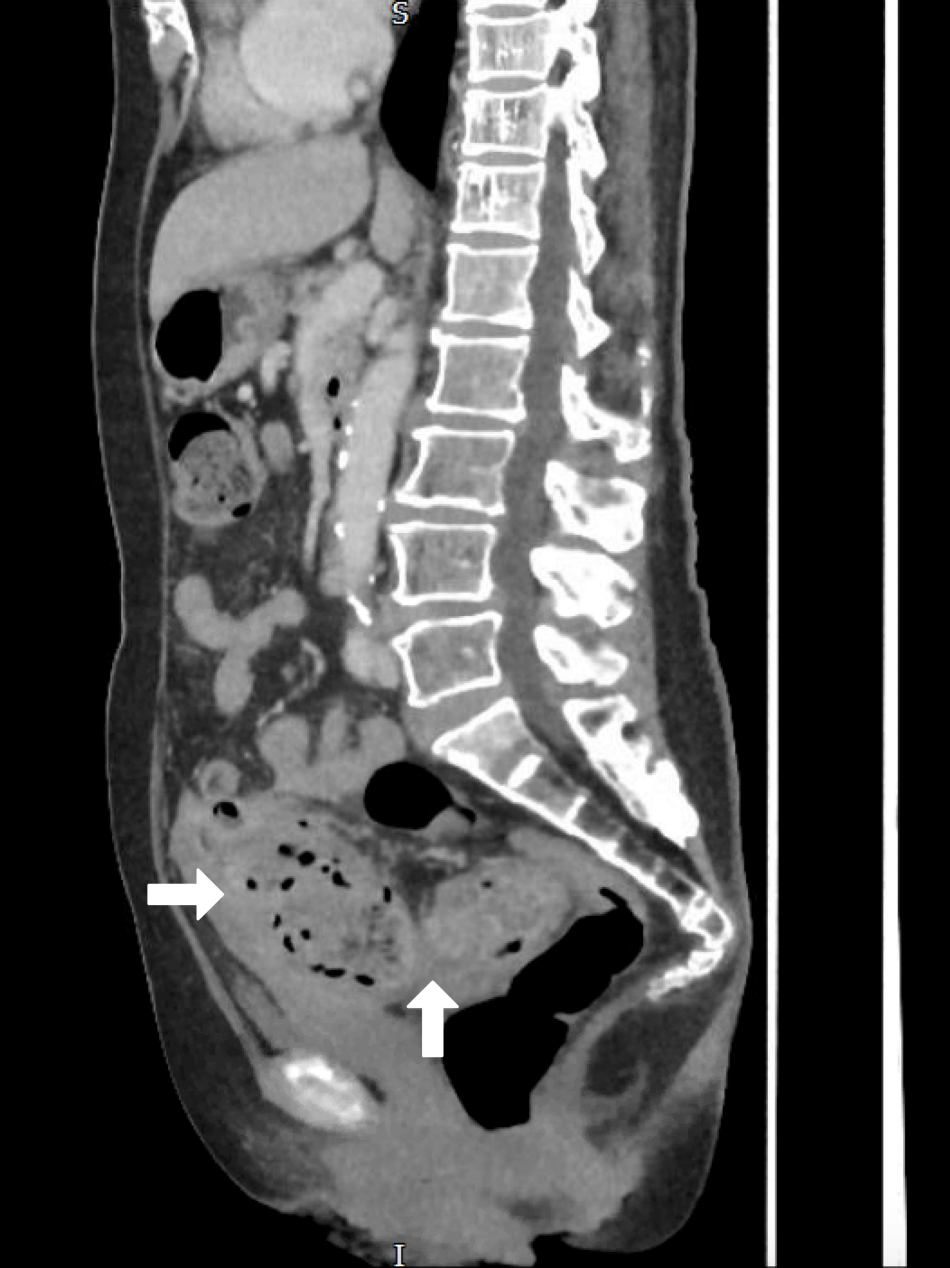
CT Abdomen & Pelvis with Contrast (Saggital View)

A 68-year-old female presented with a two-day history of generalised abdominal pain, fever, and vomiting. She had a history of poorly controlled type 2 diabetes mellitus (HbA1c: 9.2%) and obesity (BMI 37 kg/m²). On physical examination, she exhibited diffuse abdominal tenderness and guarding. Laboratory investigations revealed leukocytosis (WBC: 14,800/mm³) and elevated CRP (163 mg/L) (Table 1). CT scan confirmed Hinchey stage II perforated sigmoid diverticulitis with localised peritonitis. Also, hard impacted stool was in the sigmoid colon (Figures 1, 2, 3). 

**Table 1:** Summary of Clinical and Surgical Course

Parameter	Findings
Age/Gender	68-year-old female
BMI	37 kg/m² (Obese)
Comorbidities	Type 2 Diabetes Mellitus (HbA1c: 9.2%)
Presenting Symptoms	Generalised abdominal pain, fever, vomiting
Duration of Symptoms	2 days
Physical Examination	Tenderness and guarding in the lower abdomen
Initial Investigations	Elevated WBC (14,800/mm³), CRP 163 mg/L
Imaging Findings	CT scan showed Hinchey stage II perforated diverticulitis with localised peritonitis and impacted stool in sigmoid colon
Surgical Procedure	Emergency midline laparotomy with Hartmann’s procedure
ciNPT Protocol	Single polyurethane foam dressing with -125 mmHg suction applied intraoperatively and maintained for 5 days
Postoperative Outcome	No infection or dehiscence, complete wound healing by Day-30

**Figure 4:**
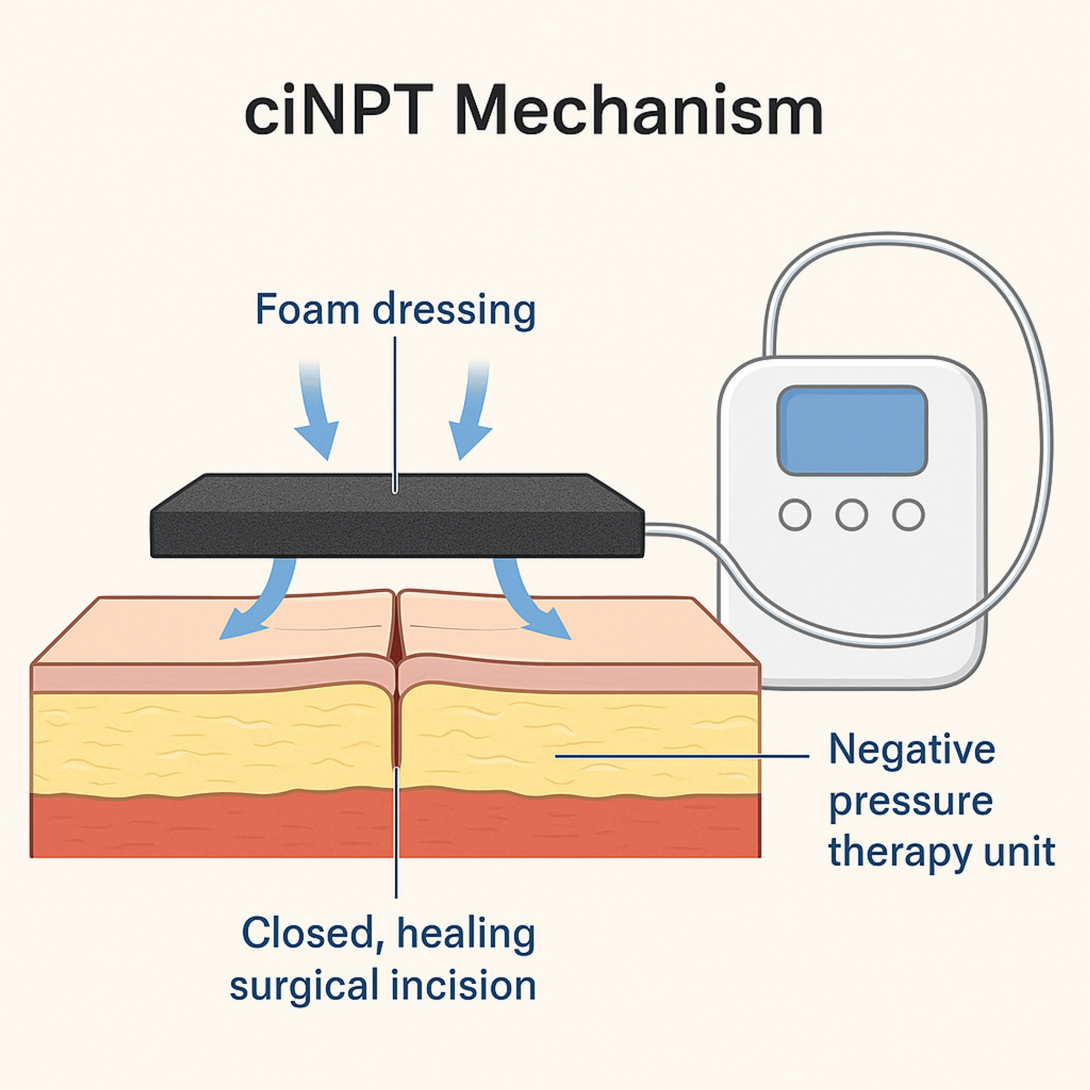
Mechanism of Closed-Incision Negative Pressure Therapy (ciNPT) Illustration showing the application of ciNPT over a closed surgical incision using a novel foam dressing connected to a negative pressure unit. Arrows indicate the directional flow of negative pressure, helping to reduce tension, improve perfusion, and protect against infection.

After initial stabilisation, the patient underwent emergency midline laparotomy and Hartmann’s procedure. Given her comorbidities and increased risk of wound complications, ciNPT was initiated intraoperatively using a novel polyurethane foam dressing system connected to a negative pressure unit set at -125 mmHg (Figure 4). A single ciNPT system was used and remained in place for five days postoperatively, without any dressing change. 

On postoperative day five, the dressing was removed. The incision was clean, dry, and intact with no signs of infection or dehiscence. The patient reported no pain or discomfort from the dressing. She was discharged on post-operative day six, and follow-up visits at day 14 and day 30 revealed a completely healed wound with no complications.

## Discussion

Patients with obesity and diabetes are particularly prone to SSIs and wound complications after abdominal surgery [3]. ciNPT is increasingly recognised as a valuable tool for reducing such risks. It offers multiple theoretical advantages: stabilising the incision, reducing lateral tension, and maintaining a closed, protected environment to prevent bacterial ingress [4].

ciNPT prevents infection by improving perfusion, reducing edema, and creating a sealed barrier that limits bacterial contamination. Additionally, continuous negative pressure removes exudate and keeps the wound environment optimal for healing [4,5].

A multicenter randomised controlled trial demonstrated a significant reduction in SSIs when ciNPT was used in obese women undergoing cesarean section (OR 0.55, 95% CI 0.31-0.97) [6]. Similarly, Sahebally et al. showed reduced infection rates and improved outcomes in colorectal surgeries when ciNPT was applied prophylactically [7].

In this case, the use of ciNPT allowed early mobilisation, no wound-related morbidity, and early discharge. These benefits align with existing literature advocating ciNPT use in general and emergency abdominal surgery [8,9].

Though further large-scale prospective studies are needed to develop formal guidelines, accumulating evidence, including this case, supports broader adoption of ciNPT in high-risk surgical patients. While ciNPT systems do have higher initial costs, these are often offset by reductions in complications, reoperations, and length of hospital stay [8].

## Conclusions

This case highlights the utility of ciNPT in preventing postoperative wound complications in high-risk emergency laparotomy patients. The successful outcome supports the growing body of evidence favouring ciNPT, especially in individuals with obesity and diabetes. Surgeons should consider its routine prophylactic use in similar scenarios.
